# Multi-omic profiling of early pregnancy small and large plasma extracellular vesicles reveals placental, metabolic, and structural adaptation signatures

**DOI:** 10.64898/2026.03.10.710888

**Published:** 2026-03-13

**Authors:** Kobe A. Abney, Taylor Hollingsworth, Alexandra Schneider, Elizabeth M. Brown, Hossein Fazelinia, Lynn A. Spruce, Rita Leite, Samuel Parry, Nadav Schwartz, Colin C. Conine, Rebecca A. Simmons

**Affiliations:** 1 Pharmacology Graduate Group, Perelman School of Medicine, University of Pennsylvania, Philadelphia, Pennsylvania, USA; 2 Center for Women’s Health and Reproductive Medicine, University of Pennsylvania, Philadelphia, Pennsylvania, USA; 3 Proteomics Core Facility, Children’s Hospital of Philadelphia, Philadelphia, Pennsylvania, USA; 4 Department of Biomedical and Health Informatics, Children’s Hospital of Philadelphia, Philadelphia, Pennsylvania, USA; 5 Department of Obstetrics and Gynecology, Perelman School of Medicine, University of Pennsylvania, Philadelphia, Pennsylvania, USA; 6 Center for Excellence in Environmental Toxicology, University of Pennsylvania, Philadelphia, Pennsylvania, USA; 7 Department of Genetics and Epigenetics Institute, Perelman School of Medicine, University of Pennsylvania, Philadelphia, Pennsylvania, USA (add epi); 8 Department of Pediatrics, Perelman School of Medicine, University of Pennsylvania, and Children’s Hospital of Philadelphia, Pennsylvania, USA

**Keywords:** Pregnancy, extracellular vesicles, placenta, maternal-fetal communication, mitochondria

## Abstract

Early human pregnancy is a critical period characterized by rapid growth and extensive maternal-fetal communication that influence maternal and fetal outcomes. Circulating extracellular vesicles (EVs) have the capacity to capture cargo that reflect these processes in real-time; however, signatures of EV subtypes during early pregnancy are poorly defined. Here we quantified mitochondrial DNA (mtDNA) and performed transcriptomic and proteomic profiling of small (~100 nm) and large (~200 nm) plasma EVs from n=10 normal pregnancies (11–15 weeks) to define subtype-specific molecular signatures. mtDNA and mitochondrial protein content were more abundant in large EVs (lEVs). lEVs also contained a more complex set of long RNAs enriched for placental, immune, and mitochondrial-related transcripts compared with small EVs (sEVs). Proteomic profiling showed enrichment of canonical EV markers and extracellular matrix proteins in sEVs, whereas lEVs were preferentially associated with pregnancy-specific proteins, including proteins related to placental hormone production. MicroRNAs (miRNAs) accounted for ~25% of small RNAs in both EV subtypes with miR-223 and miR-16 enriched in lEVs and miR-639 enriched in sEVs. These data together, support a model where small and large plasma EVs have distinct, yet complementary signatures reporting systemic adaptations during the critical 11–15 week transition period. This work establishes a foundational framework for future studies linking EV signatures to placental dysfunction and adverse outcomes.

## Introduction

The early pregnancy window between the first and second trimester (11–15 weeks) is a critical period for establishing healthy placental function and maternal adaptations as the placenta transitions from a low oxygen to a relatively higher oxygen environment. This transition is essential for determining downstream pregnancy trajectory and outcomes driven by adequate placental function. Throughout gestation, both maternal and placental cells release extracellular vesicles (EVs) into maternal circulation as a form of bidirectional communication through the delivery of nucleic acids, proteins, and metabolites (Fazeli, 2024). Accumulating evidence suggests EVs promote successful pregnancy by mediating key physiological processes such as placental metabolism, implantation, immunomodulation, spiral artery remodeling, and labor (Tannetta, 2014; Zhang, 2020; Burnett, 2016). Additionally, changes in EV concentration and cargo have been described across many pregnancy complications, highlighting the capacity of EVs to reflect maternal and fetal tissue dysfunction and potentially indicate the risk of pregnancy complications (Zhang, 2020; Barnes, 2023).

Despite this growing interest, most EV-based pregnancy studies have focused predominantly on one subpopulation, small EVs/exosomes (sEVs; ~50–150 nm), or examined bulk EV populations (Zhang 2020). However, larger vesicles (~150–500 nm), also known as microvesicles or large EVs (lEVs), remain relatively underexplored despite their potential for distinct molecular cargo and biological insights. While the biogenesis of circulating EVs cannot be directly determined, size-based fractionation supports comparative profiling of EV subtypes with different physical properties and molecular signatures. In cell culture systems, sEVs have been shown to originate predominantly from the inward budding of multivesicular bodies via endosomal sorting complexes (ESCRT) and other specialized pathways such as lipid and tetraspanin-mediated mechanisms (Lee, 2024; Trajkovic, 2008; van Niel, 2011), while lEVs typically arise from outward budding of the plasma membrane through processes regulated by lipid dynamics and cytoskeletal remodeling (Taylor, 2020; Del Conde, 2005; Muralidharan-Chari, 2009). These distinct biogenesis pathways in model systems are associated with different cargo profiles: sEVs are often enriched in small RNAs and proteins reflective of intracellular signaling pathways and molecular signatures of diseases (Valadi, 2007; Jadli, 2021; Aheget, 2020; Dogra, 2024; Byappanahalli, 2023), while lEVs reflect the functional state of the plasma membrane and cellular responses to stress or signaling cues (Spitzer, 2019; Kanada, 2015; Martellucci, 2020). Although size-fractionated circulating EVs likely represent heterogeneous populations with overlapping biogenesis origins, comparative profiling of small and large EVs provides a framework for investigating their differential molecular composition and contributions during pregnancy.

To date, no study has comprehensively compared plasma-derived small and large EVs during early gestation in healthy pregnancies across multiple molecular dimensions. In this study, we perform size-fractionated molecular profiling of circulating EVs from normal pregnancies (11–15 weeks), using a multi-omic approach focusing on four biologically informative analytes: mitochondrial cargo, large RNAs (mRNAs and long non-coding RNAs), small RNAs, and protein cargo. These analytes were selected to capture distinct and complementary signatures of systemic metabolism, cellular activity, and placental function; processes that are central to maternal adaptations and fetal development during pregnancy. By establishing a reference profile for EV subtypes early in pregnancy, this work aims to provide fundamental insight into their physiological roles and enable future studies to identify early, EV-based biomarkers of placental dysfunction and other postnatal complications.

## Methods

### Participant Cohort

The Placenta 3D study, a prospective longitudinal cohort, is an ongoing Institutional Review Board (IRB) approved study (IRB#852638) at the University of Pennsylvania. Participants are recruited from the Obstetrics & Gynecology (ObGyn) practice at the Hospital of the University of Pennsylvania (HUP). Participants presenting for a prenatal ultrasound prior to 15 weeks at 6 days’ gestation at the Maternal Fetal Medicine Ultrasound unit at HUP were screened for eligibility through medical record review. Eligible women are approached for study participation by a Clinical Research Coordinator. Inclusion criteria include a singleton pregnancy, maternal age of 18 years or older, ability to read and understand English, and willingness and ability to provide written Informed Consent. Exclusion criteria include multiple gestation and the presence of a known major fetal anomaly or genetic abnormality.

### Clinical and Demographic Data Collection

Clinical and demographic information including maternal age, gestational age at sample collection and delivery, maternal body mass index (BMI), birthweight, and race, was abstracted from the electronic medical record (Table 1). For this study, we sampled ten normal pregnancies between 11–15 weeks’ gestation uncomplicated by adverse outcomes such as preterm birth, preeclampsia, or fetal growth restriction. BMI of the women in the study ranged between 24–30 kg/m^2^, representative of the average BMI of the total cohort.

### Platelet-poor Plasma Preparation for EV Isolation

Blood was collected from a maternal peripheral vein between 11- and 15-weeks’ gestation at the first visit (V1) appointment in a K2EDTA tube. Blood was inverted gently and spun at 1000g for 10 minutes at room temperature to isolate plasma. 1 ml aliquots of plasma were transferred to 1.7 ml microcentrifuge tubes and spun at 2500g for 15 minutes at room temperature to deplete cellular debris and platelets (Małys, 2023; Coumans, 2017; Welsh, 2023). Aliquots were transferred to new 1.7 ml tubes and spun again at 2500g for 15 minutes at room temperature. The platelet-poor plasma (PPP) aliquots were transferred to cryovials (Fisherbrand, Waltham, Massachusetts, Cat # 10–500-26) and stored at −80°C.

### Isolation of Large EVs by Centrifugation

Centrifugation was performed to enrich lEVs (200–500 nm) from human PPP. For each subject, samples for mRNA analysis were derived from a 1 ml aliquot. 800 μl of a second aliquot was used for DNA isolation, and 1ml of a third aliquot was used for proteomic analysis. PPP samples were taken from −80°C storage and thawed overnight at 4°C for experiments. Using an Eppendorf 5424 tabletop centrifuge (Eppendorf, Enfield, Connecticut), plasma samples were spun at 20,000g for 30 minutes at room temperature. The supernatant was removed and transferred to a new Eppendorf tube for size exclusion chromatography. The remaining pellet (crude lEVs) was washed with 1 ml of filtered 1X PBS and centrifuged at 20,000g for 30 minutes at room temperature to remove any remaining debris. The supernatant was discarded, and the crude lEV pellet was resuspended in 78.4 μl of filtered 1X PBS for RNA extraction or 100 μl for mtDNA extraction. 15 μl of sample was aliquoted for particle counting.

### Isolation of Small EVs by Size Exclusion Chromatography and Ultracentrifugation

Size exclusion chromatography was performed to enrich sEVs using the qEV1 Gen 2 70 nm column (Izon Science, Christchurch, New Zealand) and the Automatic Fraction Collector (Izon Science, Christchurch, New Zealand) as described for plasma EVs. The supernatant collected from the first 20,000g spin was used on the column. Of the four eluted fractions, fractions 1–3 were combined and concentrated at 100,000g for 70 minutes using the Optima Max-TL Ultracentrifuge and TLA 110 rotor (Beckman Coulter, Indianapolis, Indiana) at 4°C to yield crude sEVs. The supernatant was discarded and sEVs were resuspended in 78.4 μl of filtered 1X PBS for RNA extraction or 100 μl for mtDNA extraction. 15 μl were aliquoted for particle counting.

### Nanoparticle Tracking Analysis and Cryo-electron Microscopy

EV were characterized by Nanoparticle Tracking Analysis (NTA), cryo-electron microscopy (cryo-EM), and protein surface markers per MISEV23 guidelines (Welsh, 2023). All NTA measurements were done in triplicates using the ZetaView (Particle Metrix, Inning am Ammersee, Germany) at 25°C, running on ZetaView analysis software v8.05.14 Sp7. Each EV sample was diluted in DI water directly before measurement based on manufacturer’s recommendations. lEV measurements were acquired using the following parameters: 520 nm laser, scatter filter wavelength, 25 min to 255 max brightness, tracking radius 100, camera- [Fp Sec 30, 1 cycle, frame rate 7.5], shutter 100, and sensitivity 70. sEV measurements were acquired using: 520 nm laser, scatter filter wavelength, 20 min to 255 max brightness, tracking radius 30, camera- [Fp Sec 60, 1 cycle, frame rate 30], shutter 100, and sensitivity 80. All measurements were captured in eleven fields.

For cryo-EM, 5 μl of plasma EV sample was applied onto the carbon side of the EM grid which was then blotted for 2.0 seconds and plunge-frozen into liquid ethane and imaged with a 300 keV Titan Krios G3i microscope, featuring a phase plate, K3 Summit direct detector camera, and BioQuantum GIF energy filter.

### Immunoblotting

Three lEV and three sEV crude pellets were resuspended in 30 μl of electrophoresis buffer (40 mM Tris-HCl pH 6.8, SDS 5%, 2M Thiourea, 6M Urea, 0.1 mM EDTA, blue bromo phenol traces) and incubated overnight at room temperature. CD9 expression was measured under non-reducing conditions, while Apo B100 expression was assessed under reducing conditions. The total volume (30 μl) of each sample was loaded per lane and run on Mini-Protean TGX precast 4–20% gels (Bio-Rad, Hercules, California) with Precision Plus Dual Color ladders ((Bio-Rad, Hercules, California) at 120V for 1 h. Proteins were transferred onto premium nitrocellulose membranes (Cytiva, Freiburg im Breisgau, Germany) using 200 mA over 3 h on ice. After blocking with 100% Intercept Blocking Buffer (LiCor, Lincoln, Nebraska) at room temperature for 2 h, the membranes were washed in 0.15% TBST and incubated overnight at room temperature with primary antibodies in 50% Intercept Blocking Buffer. The following primary antibodies were used: anti-CD9 (biotinylated clone HI9a, 1:1000 w/v dilution, Biolegend Cat # 312112) and anti-Apo B100 (clone C1.4, 1:1000 w/v dilution, Santa Cruz Cat # 13538). The membranes were washed in 0.15% TBST and incubated with secondary antibodies: Streptavidin (LiCor Bio Cat # 926–32230) and goat anti-mouse (LiCor Bio Cat # 926–32210) diluted 1:5000 in 0.15% TBST at room temperature for 2 h. The blots were developed by the LiCor Odyssey Imager (LiCor, Lincoln, Nebraska).

### Ethanol precipitation-based DNA extraction

Some EV-mtDNA studies have included a DNase treatment step during the DNA isolation process to degrade unencapsulated DNA. However, this step disturbs the EV corona, a dynamic layer of proteins and nucleic acids adhering to the surface of EVs, which has been shown to play an essential role in EV functionality, influencing angiogenesis, cell uptake, and immunomodulation (Wolf, 2022). Moreover, removal or disturbance of the corona, through additional purification or enzymatic treatments can significantly alter EV functions, including their uptake by recipient cells and their biological activity (Esmaeili, 2025). Therefore, here, EVs were not DNase-treated to preserve the corona and capture biologically active, EV-associated mtDNA content in its native, unstripped state.

We adapted a previously reported method used to isolate cell-free mtDNA (Ware, 2020). EV-associated mtDNA was extracted from volumes equal to 2 × 10^7^ lEVs or 1 × 10^8^ sEVs and diluted with PBS to a final volume of 37 μl. EVs were incubated at 55°C for 16 h with 1.66 μl of 10% SDS, 37.9 μl of 1 M Tris-HCl (pH 8.5), 7.6 μl of 0.5 M EDTA, 3.12 μl 5 M NaCl, 2.62 μl of 20 mg/ml Proteinase K, and 0.70 μl of 1% β-mercaptoethanol. Samples were digested for 16 h, cooled at room temperature for 5 minutes, and then diluted 4.5:1 with a premixed digestion buffer containing 0.2% SDS, 100 mm Tris-HCl, 5 mm EDTA, 200 mm NaCl based on the concentration of the stock solutions described above. Following digestion, samples were incubated for 5 minutes on ice with 170 μl of 5 M NaCl to precipitate proteins. The samples were then centrifuged at 21,000g for 20 minutes at 4°C. The supernatant was transferred to a new tube and 800 μl of cold 100% ethanol and 1 μl of GlycoBlue were added. The tubes were centrifuged again at 21,000g for 20 minutes at 4°C. The supernatant was aspirated, and the remaining pellet was washed with 500 μl of cold 70% ethanol and centrifuged at 21,000g for 20 min at 4°C. The supernatant was aspirated again, and the pellet air-dried at 37°C for 15 minutes. 60 μl of TE buffer containing 2% RNase A (200 μg/ml) was added to the tubes and incubated at 55 °C for 1 h. The resuspended DNA was aliquoted and stored at −20 °C.

### Total RNA Extraction

As previously described (Trigg and Conine, 2024), small and large EV samples were lysed in lysis buffer (6.4 M Guanidine HCl, 5% Tween 20, 5% Triton, 120 mM EDTA, and 120 mM Tris pH 8.0) with Proteinase K and incubated at 60°C for 15 minutes while shaking to extract total RNA. One volume of water and 2 volumes of TRI reagent (Invitrogen, Waltham, Massachusetts, Cat # AM9738) were added to the sample and mixed thoroughly to ensure complete lysis. The samples were transferred to a phase lock tube (discontinued; Quantabio, Beverly, Massachusetts) that was previously centrifuged empty at 20,000g for 1 minute. BCP (1-bromo-2 chloropropane) was added to the phase lock tube at 1:5 total volume and inverted 15 times to mix. The samples were centrifuged at 4°C for 4 minutes at 20,000g, and the aqueous phase was transferred to a new low-adhesion tube before adding 1 μl of Glycoblue (Invitrogen, Waltham, Massachusetts, Cat # AM9516) and 1.1 volumes of isopropanol. Samples were mixed thoroughly and incubated for 1 h at −20°C to precipitate the RNA. Following precipitation, RNA was pelleted at 20,000g for 15 minutes at 4°C and washed with freshly made, ice-cold 70% ethanol. Proceeding the wash, RNA was reconstituted in RNase-free water and incubated with DNase I for 15 minutes at room temperature. One volume of water and 2 volumes of TRI reagent were added to the sample and mixed thoroughly. The samples were transferred to phase lock tubes for phase separation and isopropanol precipitation, as described above. The final RNA pellets were reconstituted in RNase-free water for low-input mRNA library preparation.

### EV-mtDNA Measurement

EV-associated mtDNA was quantified using a TaqMan-based quantitative polymerase chain reaction (qPCR). These reactions were run in triplicate on a QuantStudio 7 real-time PCR system (Thermo Fisher Scientific, Waltham, Massachusetts). As previously described, the assay targeted mitochondria-encoded human NADH:ubiquinone oxidoreductase core subunit 1 (ND1) (Ware, 2020; Golden, 2025). Thermocycling conditions were as follows: an initial denaturation step at 95 °C for 20 s followed by 40 cycles of 95 °C for 1 s, 63 °C for 20 s, and 60 °C for 20 s.

While the CT value can indicate the relative presence of mtDNA, it does not quantify absolute copy number present in the sample. Therefore, serial dilutions of pooled human placental DNA quantified for ND1 (copies per microliter) by digital droplet PCR were used to create a standard curve (Golden, 2025). Using the standard curve, we first calculated the abundance of mtDNA present. We then normalized the abundance by the number of EVs in the starting material to estimate the number of mtDNA copies per EV (Golden, 2025). While estimating mtDNA copy number per EV is an oversimplification given the inherent heterogeneity of EV populations, this approach provides a necessary framework for direct quantitative comparison of mtDNA content between small and large EVs.

### Low-input RNA Library Prep and Next Generation Sequencing

Long RNA Libraries were prepared following an adapted protocol from single-cell RNA seq using the SMART-Seq protocol (Trigg and Conine, 2024; Trombetta, 2014). Polyadenylated RNAs from small and large EVs were reverse transcribed into cDNA using Superscript II (Invitrogen, Waltham, Massachusetts, Cat# 18064014). The cDNA was amplified with 20 PCR cycles and cleaned up with AMPure XP beads (Beckman Coulter, Indianapolis, Indiana, Cat# A63881). cDNA was then quantified with the Qubit dsDNA HS Assay Kit (Life Technologies, Waltham, Massachusetts, Cat# Q32851). Using the Nextera XT kit (Illumina, San Diego, California, Cat# FC-131–1096), a pool of uniquely indexed samples was constructed from 2 ng of the purified PCR product. A final 12 cycle amplification was performed on the pooled libraries and cleaned up using the AMPure XP beads for sequencing on a NextSeq1000.

Small RNA libraries were prepared using a modified SMARTer^®^ smRNA-Seq Kit for Illumina (Takara Bio USA, San Jose, CA) optimized for low-input small RNA species including miRNA, small interfering RNA (siRNA), PIWI-interacting RNA (piRNA), transfer RNA (tRNA), and small nucleolar RNA (snoRNA). RNA from EVs was polyadenylated and reverse transcribed into cDNA using PrimeScript RT (Takara Bio USA, San Jose, CA) with template switching to incorporate adapter sequences. To account for differences in starting material, representative samples were used to optimize PCR amplification and determine the minimum number of cycles required without overamplification. Based on this optimization, libraries from small EV RNA were amplified with 20 PCR cycles and libraries from large EV RNA were amplified with 18 PCR cycles using uniquely indexed primers. Amplified libraries were purified using AMPure XP beads (Beckman Coulter, Indianapolis, Indiana) and resolved on an 8% polyacrylamide gel. The small RNA library fraction corresponding to 171–193 bp was excised to remove adapter dimers and nonspecific products. Purified libraries were quantified with the Qubit dsDNA HS Assay Kit (Life Technologies, Thermo Fisher Scientific, Carlsbad, CA). Using the Nextera XT kit (Illumina, San Diego, CA), a pool of uniquely indexed samples was constructed from 2 ng of each purified library. The pooled libraries were prepared with a 5% PhiX spike-in and sequenced single-end on a NextSeq 1000. Adaptor sequences and 5’ adenylation from library preparation were removed using Cutadapt.

### Protein Extraction and Digestion

Small and large EV pellets were lysed in 8 M urea, 5% SDS, 100 mM Tris-HCl (pH 8), and protease inhibitors, followed by benzonase treatment. Protein concentration was estimated by in-gel Bradford staining using an E. coli lysate standard.

Proteins were digested using the S-Trap Micro protocol. Samples were reduced (TCEP), alkylated (iodoacetamide), acidified, and digested with trypsin/Lys-C. Peptides were eluted, vacuum-dried, desalted using C18 StageTips, and reconstituted in 0.1% TFA containing iRT peptides. Peptide concentration was measured at OD280 and adjusted to 400 ng/μL for injection.

### Mass Spectrometry Acquisition

Samples were randomized and analyzed on a Bruker TimsTOF HT coupled to a NanoElute2 nano-LC in DIA-PASEF mode. Peptides (800 ng) were separated on a 25 cm C18 column using a 35-minute gradient (2–30% acetonitrile). Data were acquired over an m/z range of 100–1700 with ion mobility separation (1/K0 0.70–1.30 V·s/cm^2^). DIA windows covered 299.5–1200.5 Da with a cycle time of 0.65 s. Ion mobility was calibrated using Agilent tuning mix standards.

### Proteomic Quality Control and Data Processing

Instrument performance was monitored using QuiC software with spiked-in iRT peptides. A K562 digest standard was injected before and after the sample set for system suitability assessment. Raw files were processed in Spectronaut (v20.2) using directDIA against the human UniProt reference proteome with common contaminants appended. Trypsin specificity (≤2 missed cleavages), carbamidomethylation (fixed), and N-terminal acetylation and methionine oxidation (variable) were specified. Peptide and protein FDR were controlled at 1%.

### Bioinformatic Analysis

Statistical analysis was performed using GraphPad Prism10. Outliers were determined using the ROUT method with the FDR(Q)=1% in Prism10. MtDNA samples with values below the limit of detection were excluded prior to statistical analysis. Normality of data was determined using the Shapiro Wilk test (α = 0.05). Following this, for normally distributed data an unpaired two-tailed Student’s t-test was performed to assess the difference of two means. Significance was defined as p < 0.05. Cohort demographics is presented as mean values ± standard deviation.

Long RNA data were mapped to the hg19 reference genome using Feature Counts on Dolphin Next (Yukselen, 2020) and normalized to transcripts per million (TPM). Sequencing quality control was assessed by determining the number of transcripts within each sample with TPM ≥ 1. lEV samples with less than 2,100 and sEV samples with less than 550 genes satisfying this cutoff were removed. Three lEV samples and two sEV samples did not meet this cutoff and were excluded from downstream RNA-seq analyses. Raw read counts were uploaded to R Statistical Software and using the DESeq2 package data was normalized, and differential gene expression was determined using a false-discovery rate p-value (Love, 2014). To filter out low-abundant transcripts, only genes with at least 10 counts across four samples in either group were analyzed. Differentially abundant transcripts were determined using a log_2_FC >|1|, and an adjusted p-value <0.05. Ingenuity Pathway Analysis was used for functional enrichment data analysis of DEGs.

Small RNA bioinformatic pipelines were assembled using DolphinNext (Yukselen, 2020). Read quality was assessed using FastQC, and reads were mapped sequentially to rRNA, miRbase, tRNAdb, snRNA, mRNA and Refseq using STAR and totaled using Feature counts. Mapped reads were normalized to total genome mapping reads for subsequent analysis. Small RNA differential expression was determined by a log_2_FC >|2| and an adjusted p-value <0.05 using DESeq2.

MS2 intensities were log2-transformed and median normalized in R. Proteins with complete data in at least one cohort were retained. We applied a two-level normalization strategy to ensure robust and comparable proteomics data. First, during sample preparation, all samples were processed together in a single batch and total protein concentrations were measured to normalize input amounts across samples, minimizing variability due to sample loading. Second, during data analysis, protein intensities were log2-transformed and median-centered so that each sample had a median intensity of zero. This approach reduces technical variability while preserving relative differences in protein abundance across samples. Differential abundance between groups was assessed using Limma, with significance defined as adjusted P value < 0.1 and log2FC > |1|. Proteins with statistically different abundance were prioritized for downstream bioinformatics analyses and visualized using volcano plots.

## Results

### Small and Large EVs Characterization

**Figure 1.**
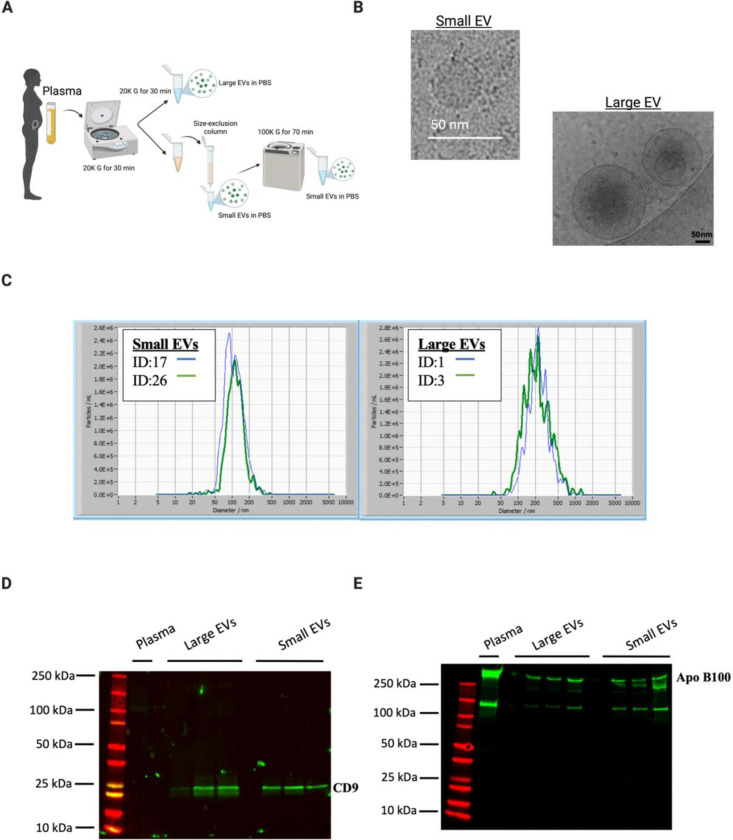
Extracellular vesicles were characterized by cryoEM, western blot, and nanoparticle tracking analysis. (A) EVs were isolated from first-trimester platelet-poor plasma via differential centrifugation and size exclusion chromatography. (B) Cryo-electron microscopy was used to visualize small and large EV morphology and size. (C) Plasma (N=1) and plasma EV samples (N=6) were immunoblotted with an antibody against CD9. (D) Plasma and plasma EV samples were immunoblotted with an antibody against lipoprotein Apo B100. (E) Nanoparticle tracking analysis was used to determine the size distribution of both small and large EVs. Graphs represent two biological samples per group. Created in BioRender. Abney, K. (2026) https://BioRender.com/l2r7gk5

Small and large EVs were isolated from the plasma of pregnant women (n=10) and characterized following the Minimal information for studies of extracellular vesicles (MISEV) criteria (Welsh, 2023) (Figure 1a). Cryo-electron microscopy showed intact vesicles with the expected morphology of small and large EVs (Figure 1b). The size distribution of both EV subtypes were confirmed by NTA, with sEVs peaking at approximately 100 nm and lEVs peaking at 200 nm (Figure 1c). EV size and concentration per sample can be found in Tables S1 and S2. Immunoblotting showed enrichment of CD9, an established EV marker, in the EV fractions compared to plasma (Figure 1d). While Apo B100, a marker of lipoprotein contamination, was not completely depleted in the EV fractions, it was significantly reduced compared to intact plasma (Figure 1e). Additional canonical EV markers such as CD63, CD81, and ALIX were confirmed through proteomic analysis. Together these data show characteristics of EVs used for downstream cargo analysis.

### Large EVs From Pregnant Women Are Associated with Higher Levels of mtDNA

**Figure 2.**
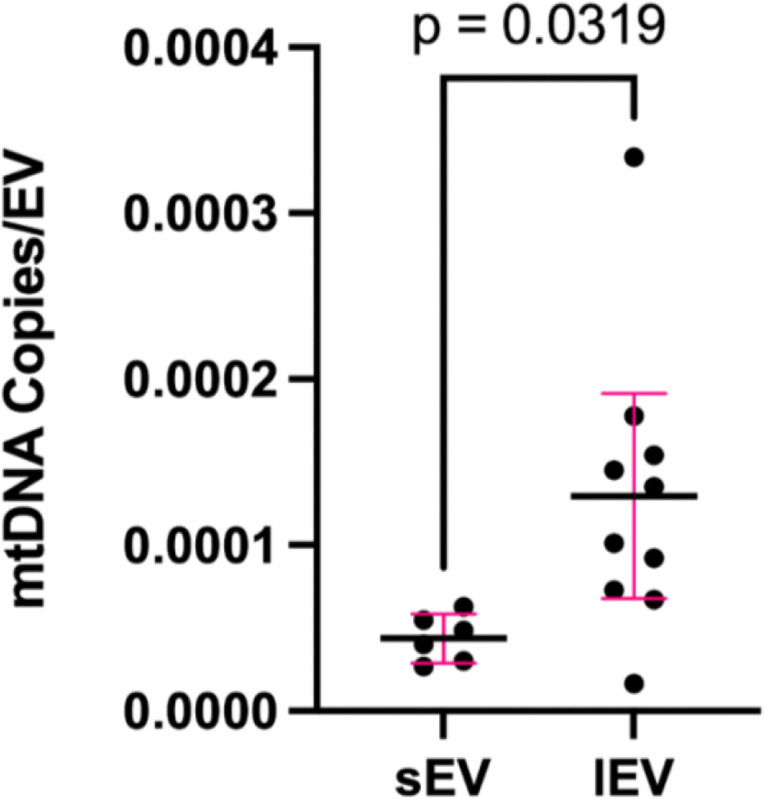
Early pregnancy large EVs are associated with more mtDNA. EV-associated mtDNA was quantified from small (N=10) and large (N=10) plasma EVs from pregnant women using qPCR. mtDNA copy number was calculated using a standard curve derived from human placenta mtDNA. An unpaired student’s t-test was performed to compare the number of mtDNA copies per small and large EV from 800 uL of platelet-poor plasma.

To date, no studies have directly quantified small and large plasma EV-associated mtDNA content in pregnancy. Therefore, we wanted to first compare the levels of EV-associated mtDNA in small and large EVs from the plasma of pregnant women (Table S3). EV-associated mtDNA was quantified from sEVs and lEVs using qPCR. Not surprisingly, we observed that the mtDNA CT values from the lEV samples were significantly lower (p<0.001) than those of the sEVs (Figure S4) indicating higher mtDNA content in lEVs. Quantifying absolute copy number, lEVs were associated with significantly more mtDNA copies compared to sEVs (Figure 2).

### Long RNAs Are More Abundant in Large EVs from Pregnant Women

To characterize transcript abundance associated with small and large EVs during early pregnancy, we isolated total RNA and sequenced the libraries of long RNAs, including messenger RNA (mRNA) and long non-coding RNA (lncRNA). While RNA delivery from sEVs is well established, characterization of EV-associated long RNAs during early pregnancy remains unexplored (Valadi, 2007; Dogra, 2024; Abdelgawad, 2025). We hypothesized that lEVs harbor long RNAs that reflect active pathways important for maternal-fetal communication, placental development, and metabolism in healthy pregnancies and that these pathways are differentially represented in small versus large EVs.

**Figure 3.**
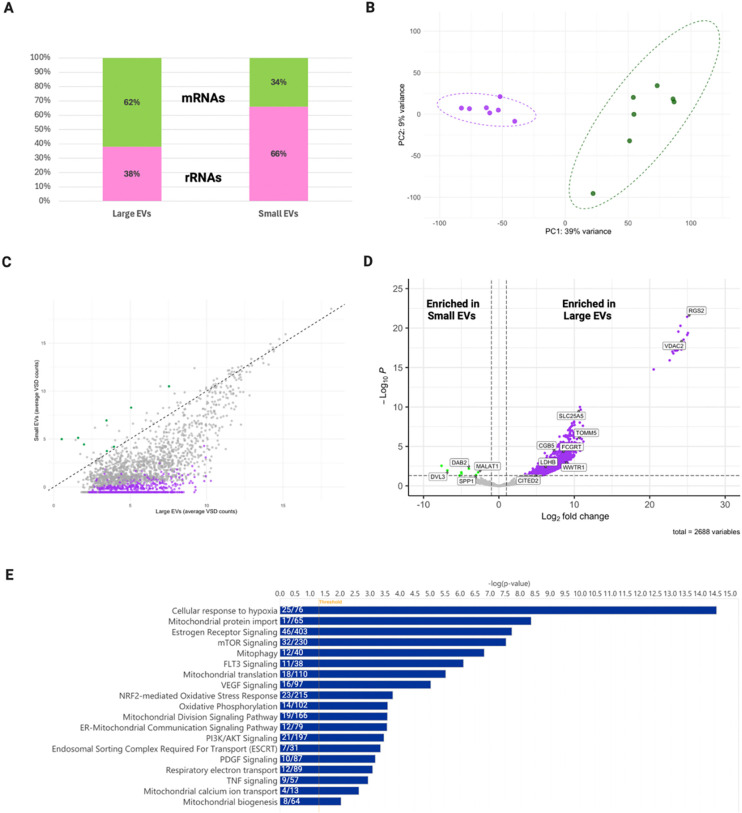
Early pregnancy large EVs are enriched with relevant long RNAs. RNA was extracted from matched small and large EVs (N=10) and long RNAs were sequenced using Next Generation Sequencing. (A) Percentage RNA class partitioning against total reads by EV subtype. Pink represents rRNAs and green represents mRNAs. (B) Principal component analysis (PCA) of the transcriptome of individual small and large EV samples isolated from maternal plasma (11–15 weeks gestation). In total, there were seven large EV samples and nine small EV samples used for downstream analysis. (C) Scatter plot of average variance stabilizing transformation (VSD) counts for large EVs (x-axis) versus small EVs (y-axis). Colored dots highlight long RNAs differentially abundant between EV subtypes. Purple indicates long RNAs enriched in large EVs and green indicates long RNAs enriched in small EVs. The grey transcripts highlight those that were not determined to be differentially abundant by DESeq2 in R. (D) Volcano plot depicting log_2_ fold change and −log_10_ adjusted *p*-value of identified transcripts in small and large EVs. Colored dots indicate differentially abundant transcripts as determined using DESeq2. Significance threshold: log_2_FC ≥ |1| and adjusted *p*-value of ≤ 0.05. (E) Pathway enrichment of the differentially abundant transcripts between small and large EVs using Ingenuity pathway Analysis. Ratio of genes in our dataset that overlap the total genes in the IPA database are represented for each pathway within the bar. Created in BioRender. Abney, K. (2026) https://BioRender.com/gaunglo

We first assessed the percentage of RNA reads mapping to mRNA and rRNA in each EV subtype to define which vesicle population captures RNAs in the circulation the most robustly. Consistent with prior reports that EV subtypes exhibit distinct RNA-class compositions, we observed that lEVs were relatively enriched for mRNAs, whereas sEVs showed a higher proportion of rRNA reads (Conley, 2017; Dellar, 2022; Fiskaa, 2016; Miranda, 2014; Sork 2018; Wei, 2017) (Figure 3a). To our knowledge, this is the first description of such long RNA-class partitioning between size-defined plasma EV subtypes in pregnancy. Principal component analysis (PCA) separated samples by EV subtype, with lEVs clustering much tighter than sEVs (Figure 3b). A scatter plot using the average variance stabilizing transformation (VSD) counts, determined by the vst function in DESeq2, highlighted the distribution of long RNA transcripts (Figure 3c) (Love, 2014). Aligning with the percent read mapping, significantly more transcripts were enriched in the lEVs compared to sEVs.

Our analysis also revealed the presence of previously reported EV reference genes (Table S11). Similar to Zhu et al (2024), GAPDH and numerous ribosomal protein (RPL and RPS) genes were present in both EV subtypes including but not limited to RPL18/A and RPS27/A. Additionally, we identified the presence of short and long RNAs described by Wei et al (2017). All three short transcripts, RPS27, RPLP2, and RPS6 were present in both EV subtypes in our dataset. We additionally identified the presence of YY1AP1 in lEVs similar to our previous study (Golden et al, 2025).

### Differentially Abundant Transcripts in Small and Large EVs Represent Relevant Pregnancy and Metabolic Pathways

Using DESeq2, we then determined differentially abundant transcripts between small and large EVs to establish EV subtype-specific signatures. Out of the total 2688 genes identified in our data, 1142 were differentially abundant with the overwhelming majority enriched in lEVs (Figure 3d)(Table S11).

Interestingly, the small set of transcripts enriched in sEVs consisted of one lncRNA, MALAT1, and protein coding transcripts, SPP1, DAB2, and DVL3. These three protein coding genes have been indicated in placenta transcriptomics (Gong, 2021). MALAT1 is expressed in many tissues but most significantly involved in fat deposition and adipogenesis (Piórkowska, 2024). More importantly, MALAT1 is well documented as highly enriched in both sEVs and the human placenta (Liu, 2021; Wang, 2024; Patel, 2018; Gonzalez, 2024). SPP1, a gene encoding a chemokine like protein secretory phosphoprotein 1, is highly expressed at the maternal-fetal interface with explicit functions early in pregnancy such as endometrial decidualization and trophoblast invasion (Pan, 2022). DAB2 is a critical mediator of extravillous trophoblast (EVT) viability and migration, and loss of function results in suppressed trophoblast and vascular smooth muscle interactions during spiral artery remodeling (Liu, 2024). DVL3 encodes the dishevelled segment polarity protein 3, a cytoplasmic protein highly expressed in invading trophoblasts and endothelial cells and Wnt signaling mediator (Sola, 2021; Sharma, 2018). This analysis validates the presence of relevant placental transcripts in sEVs.

Of the 1142 transcripts enriched in lEVs, 388 were also highly expressed in the placenta (FPKM > 10) (Table S11) (Gong, 2021). Compared to sEVs, lEVs were more abundant in CITED2, a negative transcriptional co-regulator of HIF-1a and hypoxia response (Yin, 2002). WWTR1, a Hippo pathway cofactor implicated in human cytotrophoblast progenitor self-renewal and extravillous trophoblast (EVT) differentiation, was also elevated in lEVs (Ray, 2022). Similarly, TIMP1, encoding the tissue inhibitor of metalloproteinases-1 protein, further supported the intentional representation of trophoblast-mediating transcripts (Vincent, 2015). Enrichment of CGB8, an hCG β-subunit gene, reflected endocrine signaling consistent with active trophoblast differentiation dynamics (Cocquebert, 2012). EV RNA cargo also reflected immune-interface biology as demonstrated by the enrichment of FCGRT, encoding the neonatal Fc receptor (FcRn) responsible for transplacental IgG transport in syncytiotrophoblast (Simister, 1996; Mimoun, 2020; Pyzik, 2023). In parallel with enriched placental transcripts, lEVs were enriched with protein encoding mitochondrial transcripts such as lactate dehydrogenase B (LDHB), translocase of Outer Mitochondrial Membrane 22 (TOMM22), mitochondrial ribosomal protein subunits (MRPL/S), PTEN induced kinase 1 (PINK1), voltage-dependent anion channel 1 and 2 (VDAC1/2), mitochondrial trifunctional protein alpha subunit (HADHA), succinate dehydrogenase subunits (SDH), mitochondrial complex I family (NDUF), mitochondrial malate dehydrogenase (MDH2), CHCHD10, growth hormone-inducible transmembrane protein (GHITM), and mitochondrial carrier homolog 1 (MTCH1) (Table S11). These transcripts were cross referenced with human placental transcriptomics and MitoCarta 3.0 confirming that validated placental and mitochondrial transcripts were specifically enriched in lEVs (Gong, 2021; Rath, 2021). MitoCarta is a curated literature-based mitochondrial database for human mitochondrial proteins and genes with annotations of their sub-localization and pathway associations (Rath, 2021). These results suggest robust enrichment of mitochondrial components in lEVs.

Even more interesting was a subset of transcripts that were present in six or more lEV groups but were completely absent in sEVs (Table S11). This included many stress-response transcripts such as ARNT, the β subunit of the heterodimeric protein Hif-1, RBM3, a stress response RNA-binding motif that acts independent of Hif-1 and mitochondria, ATF4, a transcriptional activator of the integrated stress response, LDHA, encoding lactate dehydrogenase A protein which converts pyruvate to lactate to maintain glycolysis in low oxygen environments, and SEPP1, encoding selenoprotein P the main transport protein for selenium to maternal and fetal tissues (Bagchi, 2024; Wellmann, 2004; Dey, 2010; Le, 2010; Duntas, 2020). 57 placental genes and 18 mitochondrial genes were included in this unique subset further emphasizing subtype specific placental and metabolic signatures (Gong, 2021, Rath, 2021).

Ingenuity Pathway Analysis (IPA) revealed significantly enriched canonical pathways (padj < 0.05) across the differentially abundant transcripts, providing a functional overview of EV-associated transcripts in early pregnancy (Figure 3e). Cellular response to hypoxia was a top enriched pathway, represented by 25 genes from our dataset. This pathway in particular is highly relevant to the context of our samples collected early in pregnancy. IPA also identified many mitochondrial pathways relating to mitochondrial function, oxidative phosphorylation (14 genes), and respiratory electron transport (12 genes). Additionally, hormone/pregnancy signaling pathways were enriched across differentially abundant transcripts including estrogen receptor signaling, which was among the top enriched pathways (46 genes), FLT3 signaling (11 genes ), and VEGF signaling (16 genes). The ESCRT pathway was another enriched pathway in our dataset (7 genes) validating the presence of EV biogenesis machinery.

### Subset of Long RNAs Are Predominately Detected in Large EVs

While DESeq2 prioritizes consistent shifts in mean expression across groups with low in-group variability, we identified a subset of genes that were predominately abundant in lEVs with limited or sporadic detection in sEVs. Although these transcripts were not classified as differentially abundant by DESeq2, their predominate detection in lEVs compared to sEVs suggest subtype-associated patterns not captured by mean-based differential testing. We defined this subset of transcripts as predominately associated if the mean normalized expression was greater than 10 counts across six lEV samples and no more than two sEVs. While there were no predominate transcripts detected in sEVs, 341 transcripts were predominately detected in lEVs (Table S11). Of those transcripts, 126 overlapped with highly enriched transcripts (FPKM > 10) found in the human placenta transcriptome (Table S11) (Gong, 2021). Of special interest are HLA-C, a classical HLA class I gene expressed by extravillous trophoblast that regulates maternal KIRs and uNK cell activity, and HLA-E, a non-classical HLA class I molecule promoting maternal-fetal immune tolerance through NK-cell inhibitory signaling (Moffett, 2006). HOPX, a marker of trophoblast progenitor cells, and EZR, a structural component of syncytiotrophoblast microvilli, was also predominately expressed in lEVs.

To investigate the presence of mitochondrial-related signatures in this subset of predominately lEV associated transcripts, we cross referenced our list again with the mitochondrial database, MitoCarta 3.0 (Rath, 2021). Out of the total 341 predominate lEV transcripts, ~16% (56/341) represented mitochondrial-related transcripts. Most of these are highly expressed in the placenta, such as solute carrier 25 gene family (SLC25), cytochrome c1 (CYC1), cytochrome c oxidases (COX), heat shock protein 10 (HSPE1), citrate synthase (CS), electron transfer flavoprotein beta subunit (ETFB), pyruvate dehydrogenase E1 beta subunit (PDHB), and peroxiredoxins (PRDX) (Table S11).

### miRNAs Are Consistently Abundant in Small and Large EVs

**Figure 4.**
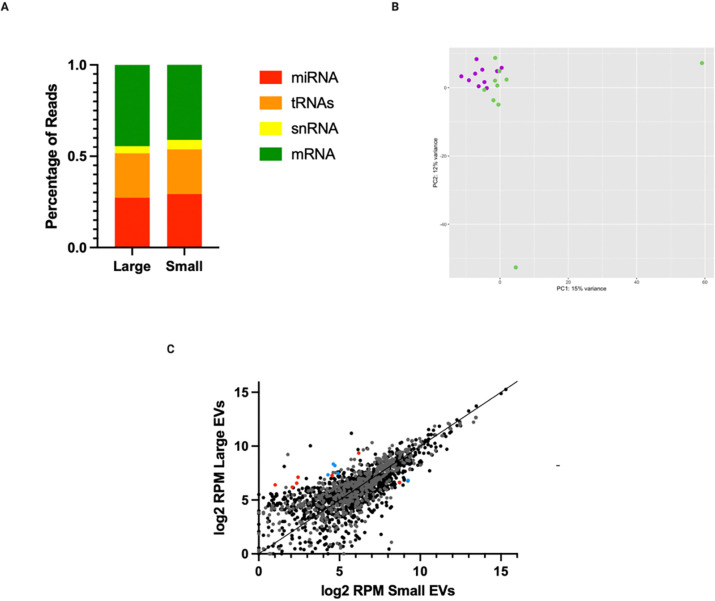
MiRNAs are consistently abundant in early pregnancy small and large EVs. Small RNAs were extracted from matched small and large EVs (N=10) and sequenced using Next Generation Sequencing. (A) Average composition of total genome small RNA reads excluding rRNAs. (B) PCA of miRNA reads only of individual small and large EV samples. Purple dots represent large EVs and green represent small EVs. (C) Scatter plot of miRNAs and tRNAs normalized to total genome reads, highlighting differentially abundant miRNAs determined by DESeq2. Red dots represent miRNAs. Blue dots represent tRNAs. Significance threshold: log_2_FC ≥ |2| and adjusted *p*-value of ≤ 0.05. Created in BioRender. Abney, K. (2026) https://BioRender.com/bev84vg

EV-miRNA has been extensively investigated in normal and complicated pregnancies (Zhang, 2020; Martin, 2026, Kothandan, 2025, Li, 2020; Menon, 2019). However, a direct comparison of small and large plasma EV small RNA composition from normal pregnancies remains limited. Here, we profiled the miRNA and tRNA composition between these two EV-subtypes (Figure S5). After rRNA removal, miRNAs and tRNAs each accounted for ~25% of reads in both small and large EVs (Figure 4a). PCA of miRNAs showed minimal clustering of small and large EVs, suggesting a similar composition (Figure 4b). lEVs were enriched with miR-16–2, miR-223, miR-361, miR-527, miR-606, miR-942, chr12.trna6-TrpCCA, chr2.trna10-SerTGA, chr2.trna21-SerTGA, and chr6.trna84-GlnTTG. In contrast, sEVs were enriched with miR-639 and chr1.trna64-GluTTC (Figure 4c). miR-16, miR-223, and miR639 have been previously reported in EV sequencing datasets (Yang, 2024; He, 2023; Huang, 2013; Cheng, 2018; Andreu, 2016; Guzman, 2016; Cruz, 2025). Predicted mRNA targets of the differentially enriched miRNAs were generated using TargetScan and subsequently analyzed using IPA to identify enriched pathways associated with these target sets (Table S12). Enrichment analysis highlighted pathways including cellular responses to hypoxia (e.g., ARNT and proteasome machinery), electron transport chain complex assembly, MTOR signaling, and gluconeogenesis among targets associated with lEV enriched miRNAs, whereas targets associated with miR-639 were enriched for tRNA and rRNA processing pathways (Table S12).

### Proteomic Analysis Revealed Robust Presence of EV Markers

**Figure 5.**
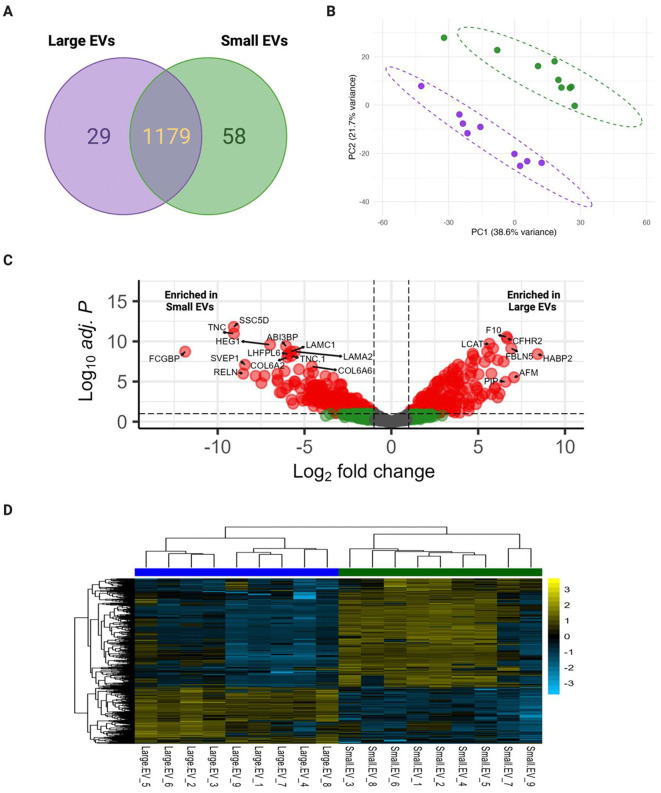
Small and large EVs have distinct proteomic profiles during pregnancy. Total protein was extracted from matched small and large EVs (N=10) and peptides were eluted for mass spectrometry analysis. (A) Venn diagram comparing protein hits between small and large EV samples.(B) Principal component analysis (PCA) of the proteome of individual small and large EV samples isolated from maternal plasma (11–15 weeks’ gestation). In total, there were nine large EV samples and nine small EV samples used for downstream analysis. (C) Volcano plot depicting log_2_ fold change and log_10_ adjusted *p*-value of identified proteins in small and large EVs. Red dots indicate differentially abundant proteins. A positive fold-change represents enrichment in lEVs, while a negative fold-change represents enrichment in sEVs. Significance threshold: log_2_FC ≥ |1| and adjusted *P*-value of ≤ 0.1. (D) Heatmap depicting the expression (log_2_ transformed) of differentially abundant proteins across EV subtypes. Created in BioRender. Abney, K. (2026) https://BioRender.com/tbotvty

To gain a comprehensive understanding of the small and large EV proteomic profile, EVs were isolated from nine out of the ten early pregnancy plasma samples for proteomic analysis. By mass spectrometry, 1266 total proteins were identified. To enhance the robustness of our data, only proteins that were quantified in at least six out of the nine samples in either group were considered for analysis. Interestingly, the analysis classified 29 proteins unique to lEVs and 58 unique to sEVs (Figure 5a)(Table S6 and S7). Using the Vesiclepedia platform, we first wanted to identify EV-specific proteins in our dataset to validate the accuracy of our EV enrichment preparations (Chitti, 2024). Out of the top 100 Vesiclepedia EV proteins, 65 overlapped with our small and large EV samples, including but not limited to CD9, CD81, and CD63 (Table S13).

### Multiple Proteins Differentially Associated with Large and Small EVs

PCA revealed the distinct clustering of large and small EVs, suggesting a unique proteomic profile between the two EV populations as previously reported (Figure 5b) (Lischnig, 2022). To better understand the components that are driving EV subtype clustering during early pregnancy, we investigated the differentially abundant proteins between the groups. 549 proteins were considered differentially abundant, with 188 being significantly abundant in lEVs and 361 being significantly abundant in sEVs (Figure 5c)(Table S13). Hierarchical clustering further illustrated the separation between EV subtypes. The heatmap visualizes relative protein abundance across all samples, revealing subtype-specific expression patterns (Figure 5d).

With regards to EV markers, we found that sEVs were enriched with top canonical EV markers, such as CD81, CD63, and ALIX, while lEVs were enriched with other EV proteins, including fibronectin 1, gelsolin, and myosin-9 (Table S8). Aligning with our immunoblot results, CD9 was also highly abundant in both EV subtypes via mass spectrometry.

We then identified differentially abundant proteins related to the placenta to reveal subtype-specific placental signatures (Table S9) (Manna, 2023). sEVs were enriched for adiponectin, syndecan-1, nidogen-1, pregnancy-associated plasma protein A, and endoglin (CD105). These proteins are associated with trophoblast basement-membrane structure, endocrine function, early placental vascular signaling, and nutrient transport (Pheiffer, 2021; Oravecz, 2022; Myatt, 2021, Romero, 2008). In contrast, lEVs were enriched for multiple pregnancy-specific glycoproteins, perlecan, collagen alpha-1(XVIII) chain, and chorionic somatomammotropin hormone 1, representing extracellular matrix components, syncytiotrophoblast-derived hormones, and maternal-fetal interface proteins (Moore, 2022; Yang, 2005; Akbas, 2020, Shi, 2020; Jeckel, 2018).

### Unique but Complementary Placental and Mitochondrial Proteins Present in Both EV Subtypes

We then cross referenced our EV proteomic data with human placenta proteomics and MitoCarta 3.0 to help distinguish true placental origin and EV signatures that may represent early indicators of trophoblast, vascular, or metabolic changes (Rath, 2022; Manna, 2023). Among the most abundant proteins associated exclusively with lEVs and relevant to the placenta (Table S13) were fibulin-5, a secreted extracellular matrix glycoprotein involved in vascular remodeling and spiral artery development (Winship, 2015; Yanagisawa, 2009; Gauster, 2011), and glycoprotein hormones alpha chain, the alpha subunit of hCG critical for placental endocrine signaling (Nwabuobi, 2017). Other proteins unique to lEVs but in lower abundance include pregnancy-specific glycoproteins 8 and 11, immunomodulators produced by trophoblasts promoting angiogenesis, trophoblast invasion, and vascular remodeling (Moore, 2022). Cytochrome b-c1 complex subunit 1, a mitochondrial respiratory-chain component important for the high metabolic demands of a developing placenta (Holland, 2018). As well as insulin-like growth factor 2, an imprinted fetal-placental growth factor that regulates nutrient uptake and placenta development (Regnault, 2022; Constância, 2002; Harris, 2011). Together, these unique lEVs proteins are associated with pathways governing trophoblast-vascular remodeling, endocrine communication, and mitochondrial energy metabolism.

Among the most abundant proteins exclusive to sEVs and relevant to the placenta were tenascin-C, an ECM glycoprotein strongly expressed in developing tissues, and laminin subunits, major trophoblast basement-membrane/ECM proteins (Table S13) (Locci, 2015; Orak, 2016; Rossi, 2025; Kuo, 2018; Liu, 2020). These unique sEV proteins highlight their potential role in shaping maternal-fetal compartment interactions.

Using MitoCarta 3.0, we then identified a panel of mitochondrial proteins, most of which were enriched in lEVs (Rath, 2021). In total, 42 mitochondrial proteins were present in our dataset. Analyzing non-differentially abundant proteins, we identified L-2-hydroxyglutarate dehydrogenase, catalase, peroxiredoxin 6, superoxide dismutase 1 in both small and large EVs (Table S13). Comparing differentially abundant proteins, choline transporter-like protein 1 was the only differentially abundant mitochondrial protein enriched in sEVs (Table S10). Examples of proteins that were in more abundant in lEVs include C18orf63 and ADP/ATP translocase 2. In contrast, there were additional mitochondria proteins that were enriched in lEVs but in lower abundance including ATP synthase F(1) complex subunit alpha, superoxide dismutase 2, thymidine kinase 2, isocitrate dehydrogenase [NADP] mitochondrial, and solute carrier family 25 member 3. These proteins collectively represent multiple nodes of mitochondrial metabolism and stress-response pathways in small and large early pregnancy EVs.

### Multi-omic Integration Reveals Concordance Across Molecular Features

**Figure 6.**
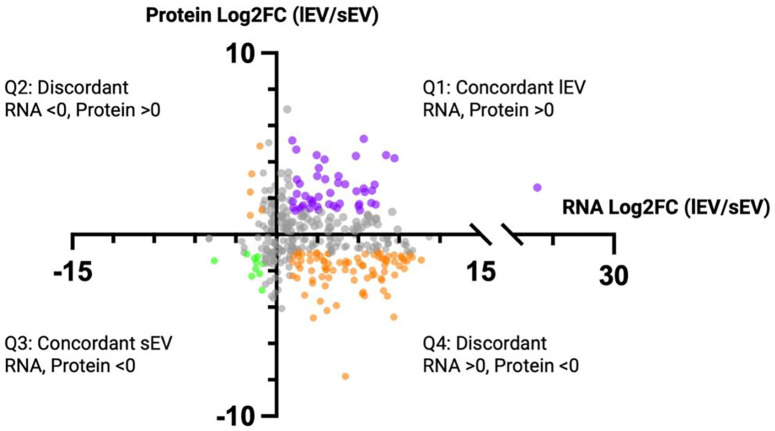
Multi-omic integration of small and large EVs reveals concordance across molecular features. A four-quadrant scatter plot of genes identified at both the transcript and protein level. Each dot represents individual genes plotted based on the average mRNA log_2_FC of lEV/sEVs (x-axis) versus the average protein log_2_FC of lEV/sEVs (y-axis). A positive fold-change represents enrichment in lEVs, while a negative fold-change represents enrichment in sEVs. Quadrant 1 indicates transcripts and proteins enriched in lEVs, with purple dots signifying a log_2_FC ≥ |1|. Quadrant 2 indicates transcripts enriched in sEVs and proteins enriched in lEVs. Quadrant 3 indicates transcripts and proteins enriched in sEVs, with green dots signifying a log_2_FC ≥ |1|. Quadrant 4 indicates transcripts enriched in lEVs and proteins enriched in sEVs. Orange dots represent discordant features with a log_2_FC ≥ |1| and grey dots represent features with a log_2_FC < |1|. Created in BioRender. Abney, K. (2026) https://BioRender.com/swbzm5i

To evaluate concordance across molecular layers, we compared transcriptomic and proteomic datasets derived from our matched small and large EV samples. We first identified genes detected at both the transcript and protein level, yielding a shared subset of 367 features (Table S14). Features were classified as concordant when transcript and protein log2 fold changes showed the same direction, whereas discordant features presented opposing directional changes. Directional agreement across features is visualized in a four-quadrant scatter plot with a significance threshold of log2FC ≥ |1| (Figure 6). Using these criteria, 47 genes demonstrated lEV concordance at the transcript and protein level including VDAC2, SOD2, SLC25A3/5, PRDX6, MDH2, and PSG1. In contrast, only 10 genes exhibited overlapping features in sEVs such as TXNIP, RDX, and S1PR1, genes previously associated with antioxidant response, cytoskeletal organization, and vascular signaling, respectively (Choi, 2023; Lee, 2021; Del Gaudio, 2020). Collectively, these observations indicate a greater number of concordant features associated with lEVs compared to sEVs.

The remaining 207 genes were discordant across both modalities, with 92 demonstrating significant magnitude differences (log2FC ≥ |1|). Among discordant features, transcript abundance more frequently associated with lEVs whereas protein abundance favored sEVs, consistent with subtype-level differences in overall transcript and protein representation observed in single-omic analyses.

## Discussion

For the first time, using size-fractionated plasma EVs and multi-omic profiling, we demonstrate that large and small plasma EVs carry distinct, but complementary molecular signatures that together reflect coordinated maternal and placental adaptation as the placenta transitions from a low oxygen to a relatively higher oxygen environment at 11–15 weeks. Our data show that lEVs are predominately enriched for mitochondrial cargo, oxygen-sensing pathway modulators, and syncytiotrophoblast-derived endocrine signaling, while sEVs are associated with extracellular matrix and basement membrane components. These differences are striking, providing a new framework for understanding how circulating EVs reflect normal placental physiology and how deviations from these signatures may indicate pathology. Elucidating the molecular mechanisms regulating early placental development is critical for identifying pathways that, when disrupted, contribute to pregnancy complications and adverse outcomes. While circulating EVs are thought to mediate maternal-fetal communication throughout gestation, how specific molecular cargo within EV subpopulations reflects placental developmental and systemic metabolic processes remains understudied.

The most pronounced distinction between EV subtypes was the enrichment of mitochondrial content associated with lEVs across multiple molecular dimensions. lEVs contained significantly higher mtDNA copy numbers compared to sEVs, representing the first direct quantification of mtDNA in size-defined plasma EVs from early pregnancy. This mtDNA enrichment was accompanied by the presence of mitochondrial transcripts and proteins suggesting a tightly regulated process. lEVs harbored significantly more mitochondrial-related mRNA transcripts and proteins than sEVs, with representation across ETC complexes, metabolite transporters, stress-response modulators, mitochondrial ribosomal proteins, import machinery, and mitochondrial assembly factors. Approximately 16% of transcripts predominantly detected in lEVs were mitochondrial related further highlighting the extensive metabolic profile of lEVs versus sEVs.

Framing these findings within the context of early pregnancy biology, the shift from glycolysis to oxidative phosphorylation supports trophoblast function, endocrine signaling, immunometabolism, and vascular remodeling as the placenta transitions to a fully functional maternal-fetal interface. Syncytiotrophoblast cells, which are highly metabolically active and interface directly with maternal blood, likely release lEVs enriched for mitochondrial cargo reflecting these adaptations. Simultaneously, maternal adipocytes, immune cells, and endothelial cells undergo significant metabolic reprogramming to support pregnancy that may be captured in circulating lEV cargo. Adipose tissue increases lipogenesis to build energy stores in preparation for the catabolic switch in the third trimester (Herrera, 2002; Jenkins, 2021). Immune cells undergo extensive metabolic reprogramming driving cellular function through a mechanism known as immunometabolism, altering cytokine production, proliferation, phagocytosis, and reactive oxygen species production to establish maternal-fetal tolerance (Jenkins, 2021; Rees, 2022). Endothelial cells increase mitochondrial biogenesis and nitric oxide production to support vascular remodeling, vasodilator production, and nutrient transport (Boeldt, 2018; Osol, 2009). The enrichment of mitochondrial biogenesis machinery, functional TCA cycle components, and quality control proteins in lEVs likely reflects these coordinated metabolic adaptations across both placental and maternal cell types.

The enrichment of mitochondrial cargo in lEVs is particularly significant given emerging evidence that mitochondrial components within EVs function as both signaling molecules and metabolically active cargo (Zhou, 2023). EV-associated mtDNA has been shown to increase under oxidative stress in trophoblasts as well as rescue metabolically impaired recipient cells (Gardner, 2024; Sansone, 2017). Moreover, mitochondrial proteins and intact mitochondria detected in EVs have been shown to incorporate into the mitochondrial network of recipient cells (Zhou, 2023; Rai, 2021; Crewe, 2021; Falchi, 2013). This suggests lEV mitochondrial cargo may serve dual roles: as molecular signatures reflecting metabolic state and as functional mediators influencing recipient cell metabolism. Given that mitochondrial dysfunction and oxidative stress are implicated in preeclampsia and fetal growth restriction (Holland, 2017; Muralimanoharan, 2012; Burton, 2009), this metabolic lEV signature provides a foundation to investigate whether circulating lEVs could serve as early biomarkers of metabolic dysfunction.

Aligning with the shift in oxygen tension, cellular response to hypoxia emerged as a top canonical pathway from our differentially abundant transcripts with HIF1 signaling as an upstream regulator. While these transcripts were measured from circulating EVs rather than only placenta-derived EVs, it has been demonstrated that a substantial amount of circulating EVs are placenta-derived (Sarker, 2014; Salomon, 2017; Nakahara, 2020). Nonetheless, we acknowledge that circulating EVs likely reflect contributions from multiple cell types, including trophoblasts, maternal immune cells, endothelial cells, and adipocytes all of which utilize HIF signaling to regulate functional adaptations during pregnancy.

The enrichment of key HIF signaling transcripts, identified by IPA, likely reflects modulation of oxygen-sensing pathways rather than indicating persistent hypoxia. These transcripts included ARNT, the constitutively expressed HIF-1β subunit required for HIF-mediated transcription, CITED2, a hypoxia-inducible negative regulator of HIF-1 activity, and proteasome machinery responsible for oxygen-dependent HIF degradation. Together, these components constitute a complete regulatory cycle of HIF activity where ARNT enables HIF transcription, CITED2 attenuates HIF transcriptional output, and the proteasome degrades HIF-1α to modulate prolonged signaling and complete this regulatory circuit. In the placental context, at 11–15 weeks, this regulatory cycle would support the shift from a low oxygen environment promoting EVT differentiation, spiral artery remodeling, and VEGF-mediated angiogenesis towards oxidative phosphorylation-dependent metabolism. Dysregulation of this transition, particularly prolonged HIF activity beyond the first trimester, has been associated with preeclampsia and fetal growth restriction (Albers, 2019; Iriyama, 2015). HIF signaling also promotes metabolic reprogramming of anaerobic glycolysis affecting macrophage, helper T cell, and regulatory T cell function necessary for maintaining maternal-fetal tolerance (Wei, 2021; O’neill and Pearce, 2016; Shi, 2011). The enrichment of VEGF signaling components, identified by IPA, alongside HIF modulators suggests the system remains engaged but controlled across different cell types, consistent with ongoing vascular maturation under regulated oxygen-sensing. These transcripts together may serve as a baseline in future studies investigating preeclampsia, fetal growth restriction, and preterm birth. For example, lEVs from women who later develop preeclampsia might show sustained HIF-1α target expression without proportionate CITED2 enrichment, or elevated mitochondrial stress markers (e.g., SOD2, PINK1) indicating oxidative damage.

In contrast to the metabolic, endocrine, and systemic signaling cargo enriched in lEVs, sEVs were preferentially enriched with structural and extracellular matrix components. While ECM proteins are essential for the function of many cell types, they are particularly important in pregnancy. Proteins uniquely or differentially abundant in sEVs included tenascin-C, laminin subunits, nidogen-1, and syndecan-1, critical components of trophoblast basement membranes and decidual extracellular matrix remodeling (Orak, 2016; Kuo, 2018; Liu, 2020). The enrichment of these proteins in sEVs, which originate from endosomal compartments involved in membrane trafficking and protein sorting, suggests these vesicles may capture cargo related to ECM organization and remodeling at the trophoblast-decidua site. It is plausible that during trophoblast differentiation and decidual remodeling at 11–15 weeks, cells at the implantation site release sEVs enriched for basement membrane and ECM proteins that reflect ongoing structural reorganization. Given that abnormal ECM remodeling and impaired trophoblast invasion are associated with preeclampsia, sEV cargo can provide insight into the adequacy of placental anchoring and invasion (Famá, 2025; Ibrahim, 2026). However, EV cell of origin resolution is needed to determine this mechanism.

Small EVs were also enriched for canonical EV markers including CD81, CD63, and ALIX, consistent with endosomal biogenesis and enrichment of tetraspanins and ESCRT machinery (Kowal, 2016; van de Wakker, 2024; Willms, 2016). This molecular signature validated distinct biogenesis pathways between EV populations and supported the interpretation that sEVs, through endosomal sorting mechanisms, are preferentially associated with proteins involved in membrane dynamics, receptor trafficking, and ECM interactions. Complementary to lEVs, sEV cargo included transcripts governing trophoblast function, although less robustly, such as SPP1, DAB2, and DVL3 which have all been associated with preeclampsia, fetal growth restriction or preterm birth (Pan, 2022; Liu, 2024; Sola, 2021).

EV-miRNA and to a lesser degree proteins are some of the most widely characterized EV cargo. In the context of pregnancy, many have associated various miRNAs with adverse pregnancy outcomes such as preeclampsia, preterm birth, gestational diabetes, and fetal growth restriction, positioning EVs as potential biomarkers (Zhang, 2020). In contrast with previous reports comparing EV subtypes, we did not find large differences in miRNA abundance between early pregnancy small and large EVs (Barranco, 2024; Varik, 2025). Of the small number of miRNAs that were differentially abundant, their predicted targets overlapped well with both single-omic associated pathways and integrated multi-omic analyses such as mitochondrial and metabolic processes, structural membrane organization, oxidative stress responses, and immunomodulation. These processes are essential to all cell types during normal physiology, especially during pregnancy. The enrichment across mRNAs, miRNAs, and proteins reinforces the role of EVs in reflecting these important mechanisms.

Collectively, our integrated multi-omic analyses support a conceptual model in which circulating small and large EVs serve distinct, yet complementary roles in maternal-fetal communication during early pregnancy. The transcriptomic and proteomic composition of lEVs captures placental responses to increasing oxygen availability, active HIF pathway modulation, mitochondrial biogenesis, trophoblast function and coordinated secretion of factors modulating maternal metabolism, immune tolerance, and vasculature. In contrast, sEVs are enriched for proteins and transcripts associated with ECM organization, basement membrane structure, and conserved EV markers, suggesting a role in shaping local uterine architecture and vascular remodeling. Rather than a single homogeneous EV pool, these findings support a division of labor where successful placental and pregnancy development during the oxygen transition requires simultaneous metabolic reprogramming, oxygen-sensing pathway modulation, endocrine maturation, structural organization, and transcriptional regulation by miRNAs. Disruption of any of these processes could lead to pathology.

An important consideration is whether EV-associated cargo functions primarily as biomarkers reporting placental and maternal state or as functional molecules influencing maternal and placental physiology. While the functional significance of EV-mRNA transfer has been debated, as most transcripts are present at low copy numbers and often fragmented, emerging evidence suggests that even low abundant, fragmented transcripts can serve important biological roles beyond translation (Dellar, 2022; Haimovich, 2017). First, 3’ UTR mRNA and small RNA fragments function as highly informative markers that reflect the transcriptional state and tissue origin of originating cells (Dogra, 2024; Abdelgawad, 2025; Dellar, 2022; Yao, 2020). In our data, we observed ~70% read distribution attributed CDS exons and ~26% attributed 3’ UTR exons, however we are limited in our abilities to determine if these are full-length or fragmented RNAs. Second, these truncated mRNAs most likely serve as regulatory molecules stabilizing, localizing, and mediating translational activity of mRNAs in target cells (Batagov & Kurochkin, 2013). Additionally, fragmented RNA species have been shown to activate immune receptors such as TLRs, further supporting functional roles beyond protein translation (Dellar, 2022; Esparza-Garrido, 2025; Schemiko Almeida, 2024).

It is also important to consider the relevance of coordinated EV cargo. While strong EV cargo concordance is not expected given inherent differences in molecule stability, detection sensitivity, and selective packaging, explicitly characterizing both cargo concordance and discordance provides insights that single-omic analysis cannot offer alone. The pathways governing protein and RNA sorting are largely independent of each other meaning concordant pairs are unlikely to arise by stochastic mechanisms. Instead, we hypothesize the co-enrichment of both transcript and protein within the same EV subtype likely reflects a regulated, and biologically relevant pathway in the originating cell. While the overlapping features from our multi-omic integration analysis revealed modest molecule-level concordance, there was convergence on shared biologically relevant themes such as mitochondrial processes, vascular signaling, and stress response. These highlight strong candidates for biomarker discovery by comparing their baseline levels in normal pregnancy versus an adverse outcome. Equally, systemic discordance should not be categorized only as technical noise but rather raise mechanistically meaningful questions regarding EV subtype-specific signatures or sorting mechanisms, originating cell activity, or pregnancy-specific cargo changes. Collectively, the integrated multi-omic framework of concordance and discordance drives conceptualization beyond single-omic analyses and supports the evaluation of EV cargo as disease-relevant biomarkers. The extent to which EVs containing various cargo work together to influence maternal physiology needs to be investigated in functional studies.

Moreover, additional limitations warrant consideration. While our sample size n=10 falls within the range of previously reported EV multi-omic studies, a larger cohort would strengthen the statistical power for biomarker validation (Rai, 2025; Hogans, 2025). Nonetheless, our matched size-fractionated design and comprehensive profiling across the same individuals enhances the confidence in our findings. Although EV enrichment was confirmed and lipoprotein contamination reduced, complete depletion of Apo B100 was not achieved. Residual lipoprotein may contribute to some detected proteins or lipids, though enrichment of canonical EV markers and vesicular morphology support confidence in EV-associated cargo. While enrichment of placenta-specific transcripts and proteins implicates substantial placental contribution to lEVs, maternal blood cells and other tissues also release EVs. However, definitive cellular origin remains constrained by technological limitations but should be considered in future studies. Single-vesicle profiling or surface marker-based enrichment could help resolve cellular origins as well. Finally, functionally testing whether sEVs and lEVs differentially influence maternal immune cell phenotypes, endothelial cell function, or trophoblast gene expression would clarify whether EVs serve solely as biomarkers or also as functional mediators. Integration of EV profiling with placental imaging, maternal metabolomics, and clinical phenotyping also could clarify how circulating EV signatures relate to placental biology and maternal physiological changes.

This study establishes the first comprehensive, multi-omic reference for small and large plasma EVs during early pregnancy, demonstrating that these populations share complementary molecular signatures reporting systemic adaptations during the critical 11–15 week transition period. Our data suggest that lEVs may function as systemic reporters of systemic metabolic reprogramming, oxygen-sensing pathway modulation, and trophoblast maturation, while sEVs reflect structural remodeling and organization in basement membrane tissues. The coordinated enrichment of mitochondrial cargo, HIF modulators, and trophoblast factors in lEVs reveals integrated adaptations essential for pregnancy success, processes whose dysregulation contributes to preeclampsia, fetal growth restriction, and other complications. By defining the molecular landscape of circulating EVs in healthy early pregnancy, this work provides a foundational reference for identifying EV-based biomarkers of placental dysfunction, detecting early deviations from normal physiology, and improving prediction and prevention of adverse pregnancy outcomes.

## Supplementary Material

Supplemental Material

Figures S1-S10 and Tables S11-S14 are available here: https://doi.org/10.5281/zenodo.18927371.

## Figures and Tables

**Table 1.** Cohort Demographics.

Variable	Control Pregnancy, n=10

**Self-Identified Race**	
**Black**	3
**White**	7

**Maternal Age-** (years)	34 (9.6)

**Gestational Age at Visit 1-** (weeks)	12.6 (0.9)

**BMI at Visit 1** ( kg/m^2^)	26.9 (2.5)

**Gestational Age at Delivery-** (weeks)	39.1 (1.0) (37–40)

**Birthweight-** (g)	3314 (448.6) (2525–4075)

Samples were collected at Visit 1 appointments (11–15 weeks). Maternal age, gestational age, birthweight, and BMI are represented as mean ±(SD). Gestational age at delivery and birthweight are followed by ranges.

## Data Availability

The RNA sequencing data reported in this study are deposited in GEO: GSE320491(mRNAseq) and GSE322470(miRNAseq). The mass spectrometry proteomics data have been deposited to the ProteomeXchange Consortium via the PRIDE partner repository with the dataset identifier PXD074792. All other data are available in the main text.
